# Early adolescent adversity alters periaqueductal gray/dorsal raphe threat responding in adult female rats

**DOI:** 10.1038/s41598-020-74457-3

**Published:** 2020-10-22

**Authors:** Mahsa Moaddab, Kristina M. Wright, Michael A. McDannald

**Affiliations:** Department of Psychology and Neuroscience, Boston College, 140 Commonwealth Ave., 514 McGuinn Hall, Chestnut Hill, MA 02467 USA

**Keywords:** Neuroscience, Learning and memory, Fear conditioning

## Abstract

Early adolescent adversity increases adult risk for anxiety disorders. The ventrolateral periaqueductal gray (vlPAG) and neighboring dorsal raphe (DR) are integral to threat prediction, and are responsive to acute stressors. Here, we tested the hypothesis that early adolescent adversity reshapes vlPAG/DR threat-related cue activity and threat probability signaling. Female, Long Evans rats experienced a battery of adverse adolescent experiences (n = 12), while controls did not (n = 8). Single-unit activity was recorded 50 + days following the final adverse experience, when the adult rats received fear discrimination consisting of danger, uncertainty and safety cues. Despite achieving fear discrimination that was equivalent to controls, vlPAG/DR threat responding was altered in adverse-experienced rats. Early adolescent adversity resulted in a greater proportion of cue-responsive neurons. Cue-excited neurons showed greater increases in firing and cue-inhibited neurons showed greater decreases. Even more, early adversity reduced flexible, threat probability signaling by cue-excited neurons and promoted more rigid, fear output signaling by cue-inhibited neurons. The results reveal long-lasting changes in vlPAG/DR threat responding resulting from early adolescent adversity.

## Introduction

Childhood adversity increases adult risk for stress and anxiety disorders^1–4^. Risk increases linearly with the number of adversity categories experienced^2,5–7^, with children experiencing 4 + categories at greatest risk. A contemporary view of the link between early adversity and adult psychiatric disorders is of latent vulnerability^8^. Early adversity reshapes the function of core neural circuits underlying fundamental behavioral processes^9^.

The amygdala and the periaqueductal gray are core components of a neural circuit for fear^10–12^. In the historical view, amygdala-generated threat probability signals are relayed to the periaqueductal gray to organize fear output^13–15^. Due to its posited role in integration, altered amygdala threat processing has been offered as a candidate for latent vulnerability^16–18^. For example, exaggerated amygdalar responses to negative facial expressions are observed in adults that were maltreated as children, but do not show overt differences in behavior and are free of psychiatric disorders^19^.

The periaqueductal gray has not been viewed as a neural locus for latent vulnerability, perhaps due to its posited role in fear output. Previous work from our laboratory recorded single-unit activity in the ventrolateral periaqueductal gray (vlPAG) while rats discriminated cues predicting unique foot shock probabilities: danger (p = 1.00), uncertainty (p = 0.375), and safety (p = 0.00). Activity patterns of cue-excited vlPAG neurons^20–23^ reflected the foot shock probability associated with each cue (threat probability), rather than fear output^24^. Activity patterns of cue-inhibited neurons showed signaling of fear output and threat probability^25^. The vlPAG may then serve a more integrative role in fear^26–28^, marking disruptions in periaqueductal threat function as a candidate for adversity-induced latent vulnerability. Using this same procedure, we have revealed an essential role for the dorsal raphe (DR), a neighboring periaqueductal region, in threat prediction^29^.

Our laboratory has developed an early adversity procedure in rats consisting of multiple adversity types, capturing a key feature of people at highest risk for psychiatric disorders. Post-weaning, pre-pubertal rats receive four adversity types, five times each, over ten consecutive days: cold swim, restraint, tail pinch and cat hair exposure^30,31^. Each adverse experience has been shown to induce vlPAG activity^32–35^. More than 50 days since the final adverse experience, adult rats are tested in fear discrimination procedure consisting of danger, uncertainty and safety cues. Here, we combined this behavioral approach with awake-behaving, single-unit recording to determine if early adversity reshapes vlPAG/DR responding to threat in adult, female rats.

## Materials and methods

The periaqueductal recording-fear discrimination approach is based on prior work from our laboratory^24,25,36^.

### Experimental subjects

Subjects were 20 female Long Evans rats born in the laboratory. Six Long Evans dams (Charles River Laboratories, Wilmington, MA) arrived at the laboratory on gestational day 14–16. Pups were born in the Boston College Animal Care Facility, housed with mothers until postnatal day (P) 21, when they were weaned, sexed, and then singly housed throughout the duration of the experiment. All rats were maintained on a 12 h light cycle (lights off at 6:00 pm) and received food (standard laboratory chow, 18% Protein Rodent Diet #2018, Harlan Teklad Global Diets, Madison, WI) and water ad libitum. Rats were weighed three times per week starting on P24 until P55 to track physical development. Starting on P56, rats were maintained at 85% of their free-feeding body weight except during surgery and post-surgery recovery periods where animals had ad libitum access to food. All protocols were approved by the Boston College Animal Care and Use Committee, and all experiments were carried out in accordance with the NIH guidelines regarding the care and use of rats for experimental procedures.

### Early adolescent adversity

**Figure 1:**
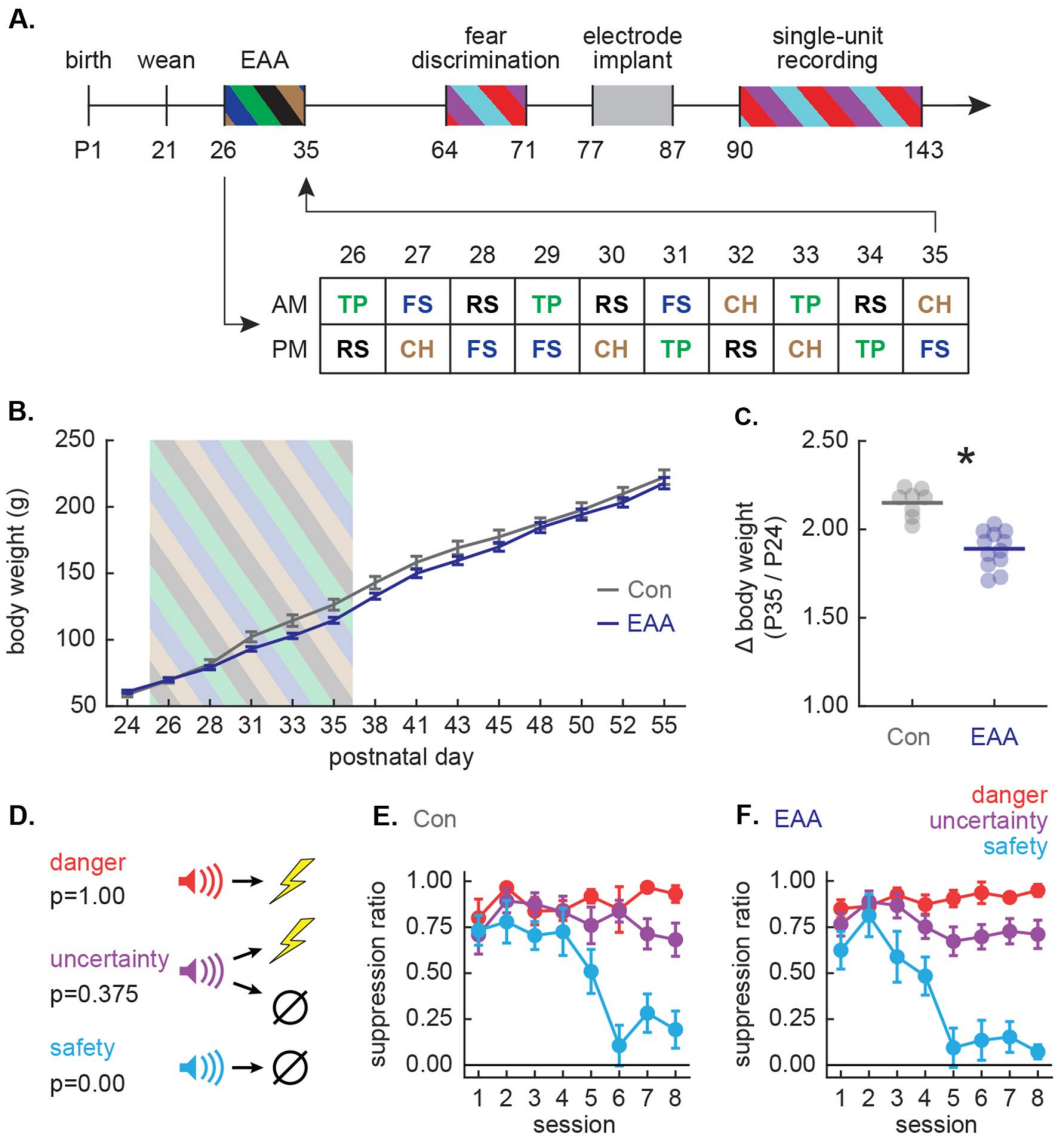
Experiment outline, adolescent body weight and adult fear discrimination. (**A**) Female, Long Evans rats were born in the lab and weaned on postnatal day (P) 21. From P26 to P35, 12 rats underwent early adolescent adversity (EAA) procedure. EAA procedure consisted of morning (AM) and afternoon (PM) exposures to one of four stressors: tail pinch (TP, green), restraint stress (RS, black), forced cold water swim (FS, dark blue), or cat hair exposure (CH, brown). The exact order of stressor presentation over the 10 consecutive exposure days is shown. Control rats (Con, n = 8) were handled each day but did not receive stressors. In adulthood (P64–P71), Con and EAA rats underwent eight sessions of Pavlovian fear discrimination. From P77 to P87, all rats were implanted with drivable microelectrode bundles dorsal to the vlPAG. Following recovery, rats were returned to fear discrimination and single-unit activity was collected from ~ P90 to P143 at the latest. (**B**) Mean ± SEM body weight (g) is shown for Con (gray) and EAA (dark blue) rats from P24 to P55. Striped box indicates the beginning (P26) and end (P35) of EAA procedure. (**C**) Individual data points and mean (indicated by horizontal line) for change in body weight (P35/P24) in Con and EAA. *Significant difference between groups (t_18_ = 6.11, p = 9.00 × 10^–6^; independent samples *t* test). (**D**) Pavlovian fear discrimination consisted of three distinct auditory cues, each predicting a unique probability of foot shock: danger (p = 1.00, red), uncertainty (p = 0.375, purple), and safety (p = 0.00, blue). (**E**, **F**) Mean ± SEM suppression ratios to danger, uncertainty, and safety cues are shown for the initial 8 discrimination sessions for (**E**) Con and (**F**) EAA rats.

From P26 to P35, early adolescent adversity (EAA) rats (n = 12) received twice daily adverse experiences, while non-exposed rats served as Controls (n = 8). Each EAA rat experienced four adverse experiences, five times each, for a total of 20 events (see Fig. 1A). Each day, the first adverse experience began at ~ 9:00 am, and the second began at ~ 3:00 pm. Adverse experiences included forced cold water swim, tail pinch, cat hair exposure, and restraint stress. Because adversity procedures were performed in the housing room, control and EAA rats were housed in separate rooms during the adversity procedures. Previous research has shown that male, but not female, experimenters induce additional stress, and a t-shirt worn by a male has the same effect as a male present in the room^37^. Therefore, during each adverse experience, a machine-washed t-shirt that had been slept in overnight by a male experimenter was present in the room in order to control for experimenter sex. A female experimenter was always present during adversity procedures. Two weeks after the conclusion of adversity (P49), EAA rats were moved into the colony room with the Control rats for the remainder of the experiment.

#### Forced swim

EAA rats were placed in a clear 10-L plastic cylinder filled with 10 °C water for 5 min. The cylinder was filled such that the rats were unable to touch the bottom or reach the top. Upon the conclusion of the 5 min, rats were immediately removed from the water and thoroughly dried with a towel before placement back in the home cage.

#### Tail pinch

EAA rats were placed in an empty, clear plastic mouse cage with a micro-isolator lid. A half-inch binder clip was placed on the base of the tail for 5 min. Upon the conclusion of 5 min, the binder clip was immediately removed, and each rat was placed back in the home cage.

#### Cat hair exposure

EAA rats were placed in an empty, clear plastic mouse cage with a wire top and micro-isolator lid. A ball of cat hair was suspended via a hair net secured to the wire top of the cage. The cat hair was obtained from three cats that were certified disease-free by a veterinarian. Rats were placed in the cage with cat hair for 5 min then immediately placed back in the home cage.

#### Restraint

EAA rats were placed in a clear plastic restraint tube (2″ diameter flat bottom restrainers, Braintree Scientific, Braintree, MA) for 30 min. Upon the conclusion of 30 min, rats were immediately removed from the tube, and each rat was placed back in the home cage.

### Electrode assembly

Microelectrodes consisted of a drivable bundle of sixteen 25.4 µm diameter Formvar-Insulated Nichrome wires (761500, A-M Systems, Carlsborg, WA) within a 27-gauge cannula (B000FN3M7K, Amazon Supply) and two 127 µm diameter PFA-coated, annealed strength stainless-steel ground wires (791400, A-M Systems, Carlsborg, WA). All wires were electrically connected to a nano-strip omnetics connector (A79042-001, Omnetics Connector Corp., Minneapolis, MN) on a custom 24-contact, individually routed and gold immersed circuit board (San Francisco Circuits, San Mateo, CA). Sixteen individual recording wires were soldered to individual channels of an Omnetics connector. The sixteen wire bundle was integrated into a microdrive permitting advancement in ~ 42 μm increments.

### Surgery

From P77 to P87, stereotaxic surgery was performed aseptic conditions under isoflurane anesthesia (1–5% in oxygen). Carprofen (5 mg/kg, i.p.) and lactated ringer’s solution (10 mL, s.c.) were administered preoperatively. The skull was scoured in a crosshatch pattern with a scalpel blade to increase efficacy of implant adhesion. Six screws were installed in the skull to further stabilize the connection between the skull, electrode assembly and a protective head cap (screw placements: two anterior to bregma, three between bregma and lambda ~ 3 mm medial to the lateral ridges of the skull, and one on the midline ~ 5 mm posterior of lambda). A 1.4 mm diameter craniotomy was performed to remove a circular skull section centered on the implant site and the underlying dura was removed to expose the cortex. Nichrome recording wires were freshly cut with surgical scissors to extend ~ 2.0 mm beyond the cannula. Just before implant, current was delivered to each recording wire in a saline bath, stripping each tip of its formvar insulation. Current was supplied by a 12 V lantern battery and each Omnetics connector contact was stimulated for 2 s using a lead. Machine grease was placed by the cannula and on the microdrive.

For implantation dorsal to the vlPAG, the electrode assembly was slowly advanced at a 20° angle to the following coordinates from cortex (anterior–posterior: − 8.00 mm, medial–lateral: − 2.45 mm and dorsal–ventral: − 5.12 mm). Once in place, stripped ends of both ground wires were wrapped around the two most posterior screws inserted previously to ground the electrode. The microdrive base and a protective head cap surrounding the electrode assembly were cemented in place at the end of the procedure using orthodontic resin (C 22-05-98, Pearson Dental Supply, Sylmar, CA), and the Omnetics connector was affixed to the head cap.

### Behavior apparatus

The apparatus for Pavlovian fear conditioning consisted of two individual chambers with aluminum front and back walls retrofitted with clear plastic covers, clear acrylic sides and top, and a grid floor. Each grid floor bar was electrically connected to an aversive shock generator (Med Associates, St. Albans, VT) through a grounding device. This permitted the floor to be grounded at all times except during shock delivery. An external food cup and a central nose poke opening, equipped with infrared photocells were present on one wall. Auditory stimuli were presented through two speakers mounted on the ceiling.

### Nose poke acquisition

Prior to discrimination sessions, on P56, rats were food-deprived to 85% of their free-feeding body weight and were fed specifically to maintain this weight through the behavioral procedure. Starting on P58, rats were shaped to nose poke for pellet (Bio-Serv, Flemington, NJ) delivery in the experimental chamber using a fixed ratio schedule in which one nose poke yielded one pellet. Shaping sessions lasted 30 min or until approximately 50 nose pokes were completed. Over the next 5 days, rats were placed on variable interval (VI) schedules in which nose pokes were reinforced on average every 30 s (VI-30, day 1), or 60 s (days 2 through 5). For the remainder of behavioral testing, nose pokes were reinforced on a VI-60 schedule independent of all Pavlovian contingencies.

### Fear discrimination

Prior to recording (P64–P71), each rat received eight, 93-min sessions of fear discrimination. Each session consisted of 32 trials, with a mean inter-trial interval of 3.5 min. Auditory cues were 10 s in duration and consisted of repeating motifs of a broadband click, phaser, or trumpet (listen or download: https://mcdannaldlab.org/resources/ardbark). Each cue was associated with a unique probability of foot shock (0.5 mA, 0.5 s): danger, p = 1.00; uncertainty, p = 0.375; and safety, p = 0.00. Auditory identity was counterbalanced across rats. Foot shock was administered 2 s following the termination of the auditory cue on danger and uncertainty shock trials. This was done in order to observe possible neural activity during the delay period is not driven by an explicit cue. A single session consisted of six danger trials, ten uncertainty no-shock trials, six uncertainty shock trials, and ten safety trials. The order of trial type presentation was randomly determined by the behavioral program, and differed for each rat, each session. After the eighth session, rats were removed from discrimination, given full food and received stereotaxic surgery. Following recovery, discrimination (identical to that described above) resumed with single-unit recording. The microelectrode bundles were advanced in ~ 42–84 μm steps every other day to record from new units during the following session.

### Single-unit data acquisition

During recording sessions, a 1 × amplifying headstage connected the Omnetics connector to the commutator via a shielded recording cable (Headstage: 40684-020 and Cable: 91809-017, Plexon Inc., Dallas TX). Analog neural activity was digitized and high-pass filtered via amplifier to remove low-frequency artifacts and sent to the Ominplex D acquisition system (Plexon Inc., Dallas TX). Behavioral events (cues, shocks, nose pokes) were controlled and recorded by a computer running Med Associates software. Timestamped events from Med Associates were sent to Ominplex D acquisition system via a dedicated interface module (DIG-716B). The result was a single file (.pl2) containing all time stamps for recording and behavior. Single-units were sorted offline with a template-based spike-sorting algorithm (Offline Sorter V3, Plexon Inc., Dallas TX). Timestamped spikes and events (cues, shocks, nose pokes) were extracted and analyzed with statistical routines in Matlab (Natick, MA).

### Histology

Rats were deeply anesthetized using isoflurane and final electrode coordinates were marked by passing current from a 6 V battery through 4 of the 16 nichrome electrode wires. Rats were transcardially perfused with 0.9% biological saline and 4% paraformaldehyde in a 0.2 M Potassium Phosphate Buffered Solution. Brains were extracted and post-fixed in a 10% neutral-buffered formalin solution for 24 h, stored in 10% sucrose/formalin and sectioned via microtome. All brains processed for light microscopy using anti-tryptophan hydroxylase immunohistochemistry (T8575, Sigma-Aldrich, St. Louis, MO) and a NovaRed chromagen reaction (SK-4800, Vector Laboratories, Burlingame, CA). Sections were mounted, imaged using a light microscope (Axio Imager Z2, Zeiss, Thornwood, NY) and electrode placement was confirmed^38^.

### Statistical analysis

#### Calculating suppression ratios

**Figure 2:**
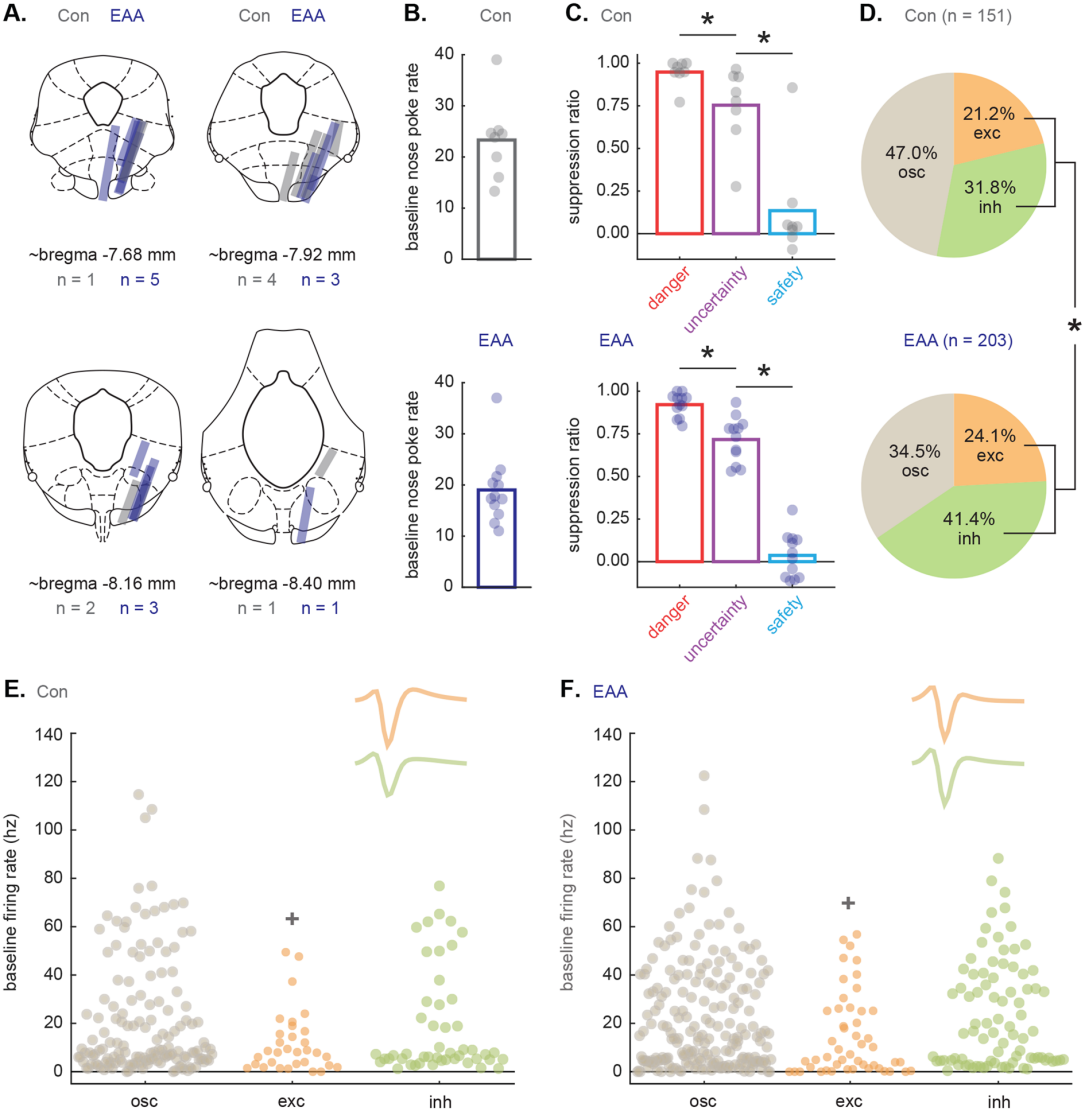
Histology, fear discrimination and single-unit characteristics. (**A**) Histological reconstruction of microelectrode bundle placements in vlPAG/DR for Con (n = 8, gray), and EAA (n = 12, dark blue) rats during recording sessions. (**B**) Mean (bar) and individual (data points) baseline nose poke rate are shown for each Con (gray, top) and EAA (dark blue, bottom) rat. (**C**) Mean (bar) suppression ratio for danger (red), uncertainty (purple), and safety (blue) trials is shown for all sessions in which single-units were recorded in Con and EAA rats. Data points for each subject are superimposed on group means for Con (gray, top) and EAA (dark blue, bottom). *Significant difference between each cue pair (danger vs. uncertainty; uncertainty vs. safety) for Con and EAA (all t > 3.00, all p ≤ 0.01; paired samples *t* tests). (**D**) Pie charts show the proportion of neurons that were cue-excited (exc, orange), cue-inhibited (inh, green) or fell outside selection criteria (osc, gray) for Con (n = 151, top) and EAA (n = 203, bottom). *Significant difference between groups (χ^2^ = 5.68, p = 0.02; Chi-square test). (**E**, **F**) Baseline firing rate for outside selection criteria (osc), cue-excited (exc) and cue-inhibited (inh) neurons from (**E**) Con and (**F**) EAA rats are shown. ^+^Significant main effect of functional type (F_2,346_ = 7.35, p = 0.001; ANOVA). Mean waveform is shown for cue-responsive neurons (exc, inh) from Con and EAA rats. Colors maintained from (D).

Fear was measured by suppression of rewarded nose poking, calculated as a ratio: [(baseline poke rate – cue poke rate)/(baseline poke rate + cue poke rate)]^39–44^. The baseline nose poke rate was taken from the 20 s prior to cue onset and the cue poke rate from the 10 s cue period. Suppression ratios were calculated for each trial using only that trial’s baseline. A ratio of ‘1’ indicated high fear, ‘0’ low fear, and gradations between intermediate levels of fear. Suppression ratios were analyzed with analysis of variance (ANOVA) with factors of group, cue and session (Fig. 1E, F); group and cue (Fig. 2C).

### Body weight

Body weight (g) was taken Monday, Wednesday and Friday from weaning to adulthood. Body weight was analyzed with ANOVA with factors of postnatal day and group (Fig. 1B). Change in body weight resulting from early adversity procedure was calculated by dividing body weight on P35 (final day of early adversity procedure) by body weight on P24 (Fig. 1C).

### Identifying cue-responsive neurons

**Figure 3:**
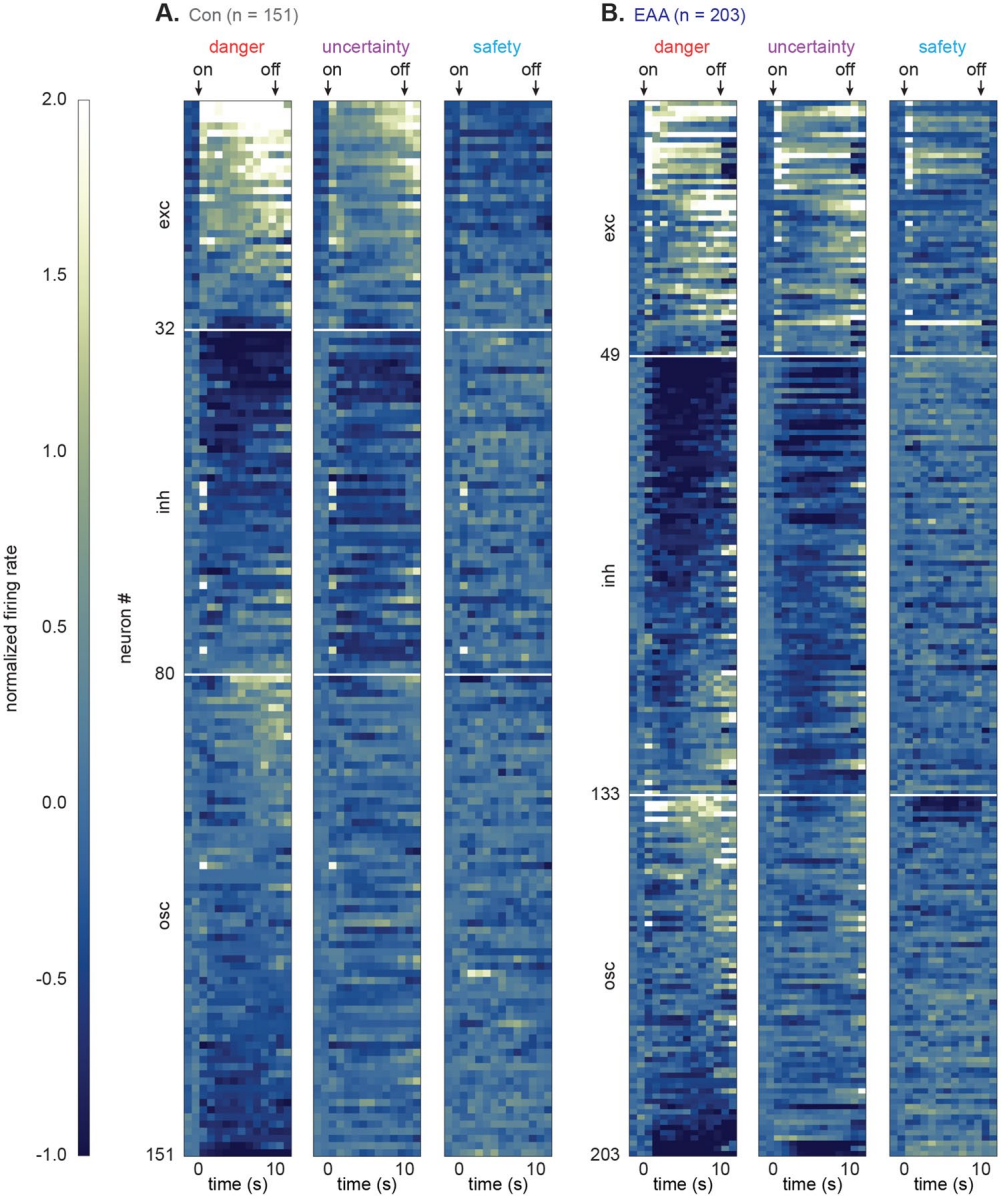
Heat plot for Control and EAA single-units. (**A**, **B**) Heat plots for normalized firing rate (Z score) to danger (red, left), uncertainty (purple, middle), and safety (blue, right) from all neurons from (**A**) Con (n = 151) and (**B**) EAA (n = 203) subjects. Cue onset (on) and offset (off) are indicated by black arrows. Each line represents an individual neuron. Neurons from each subject group are split into cue-excited (exc, top), cue-inhibited (inh, middle) and neurons outside the selection criteria (osc, bottom). Color scale for normalized firing rate is shown to the left; lighter colors indicate increased firing over baseline and darker colors decreased firing under baseline.

Single-units were screened for cue responsiveness by comparing raw firing rate (Hz) during the 10 s baseline period just prior to cue onset and during the first 1 s cue interval or the final 5 s cue interval. A *t* test was performed for each of the three cues (danger, uncertainty and safety), corrected for six comparisons (p < 0.0083). A neuron was cue-excited if it significantly increased firing over baseline to at least one of these six epochs, but did not significantly decrease firing during an epoch. A neuron was cue-inhibited if it significantly decreased firing from baseline to at least one of these six epochs, but did not significantly increase firing during an epoch. Neurons outside of the selection criteria were shown in Fig. 3, but were not further analyzed. The proportion of cue-responsive units obtained from Control and EAA rats was compared using the Chi-square test (Fig. 2D): https://www.socscistatistics.com/tests/chisquare/default2.aspx.

### Z score normalization

For each neuron, and for each trial type, firing rate (Hz) was calculated in 250 ms bins from 20 s prior to cue onset to 20 s following cue offset, for a total of 200 bins. Mean firing rate over the 200 bins was calculated by averaging all trials for each trial type. Mean differential firing was calculated for each of the 200 bins by subtracting mean baseline firing rate (2 s prior to cue onset), specific to that trial type, from each bin. Mean differential firing was Z score normalized across all trial types within a single neuron, such that mean firing = 0, and standard deviation in firing = 1. Z score normalization was applied to firing across the entirety of the recording epoch, as opposed to only the baseline period, in case neurons showed little/no baseline activity. As a result, periods of phasic, excitatory and inhibitory firing contributed to normalized mean firing rate (0). For this reason, Z score normalized baseline activity can differ from zero. Z score normalized firing was analyzed with ANOVA using group, bin and trial type as factors. F and p values are reported, as well as partial eta squared (η_p_^2^) and observed power (op).

### Population and single-unit firing analyses

**Figure 4:**
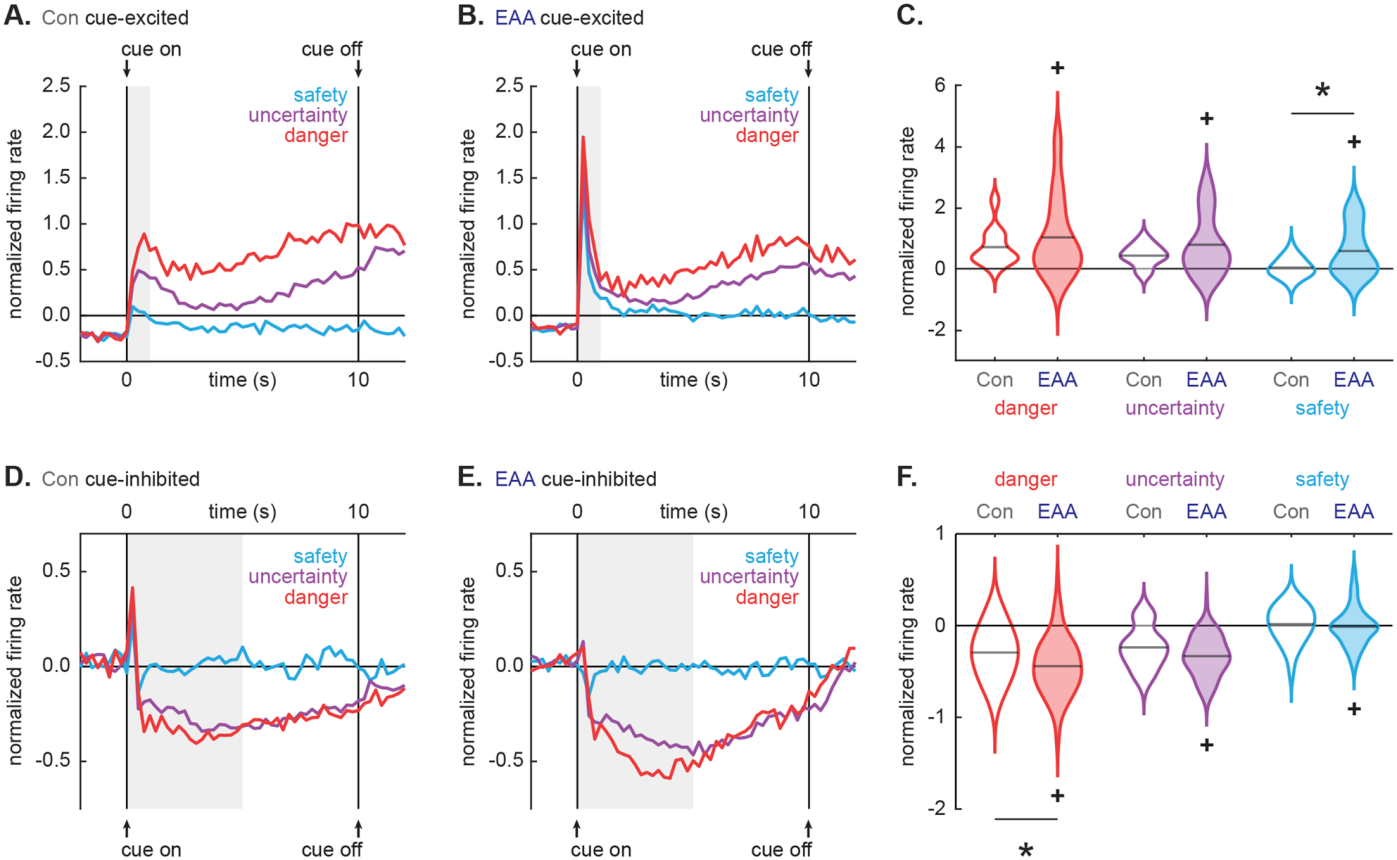
Inflated cue responses in EAA single-units. (**A**, **B**) Mean normalized firing rate (Z score) to danger (red), uncertainty (purple) and safety (blue) is shown for the 2 s pre-cue period, the 10 s cue period, and 2 s post-cue period for (**A**) Con cue-excited (n = 32) and (**B**) EAA cue-excited (n = 49) neurons. Cue onset (on) and offset (off) are indicated by vertical black lines. Light gray boxes indicate interval analyzed in (**C**). (**C**) Violin plot of normalized firing rate during the first 1 s cue interval, is shown for each cue for the cue-excited population in Con (open) and EAA (closed). Mean normalized firing rate for each cue is indicated by gray horizontal line. *Significant differential firing to safety between Con and EAA (t_79_ = 3.61, p = 0.001, Bonferroni-corrected, independent samples *t* test). ^+^Significant main effect of group (F_1,79_ = 4.96, p = 0.03; ANOVA). (**D**, **E**) Normalized firing for (**D**) Con cue-inhibited (n = 48) and (**E**) EAA cue-inhibited (n = 84) neurons plotted as in (**A**) and (**B**). Light gray boxes indicate interval analyzed in (**F**). (**F**) Violin plot of normalized firing rate during the first 5 s cue interval, is shown for each cue for the cue-inhibited population in Con (open) and EAA (closed). *Significant differential firing to danger between Con and EAA (t_130_ = 2.70, p = 0.008; Bonferroni-corrected, independent samples *t* test). ^+^Significant main effect of group (F_1,130_ = 10.82, p = 0.001; ANOVA).

Population firing was analyzed using ANOVA with group, trial type and bin (250 ms) as factors (Fig. 4A, B, D, E). ANOVA for cue firing contained three trial types (danger, uncertainty and safety). Uncertainty trial types were collapsed because they did not differ for either suppression ratio or firing analysis. This was expected, during cue presentation rats did not know the current uncertainty trial type. F statistic, p value, partial eta squared (η_p_^2^) and observed power (op) are reported for effects and interactions. ANOVA was also performed for the first 1 s of firing for cue-excited neurons (Fig. 4C) and for the first 5 s of firing for cue-inhibited neurons (Fig. 4F). Differential firing to each cue between the groups was compared using independent sample *t* test, corrected for three comparisons. The distribution of single-unit firing was visualized using violin plots (https://www.mathworks.com/matlabcentral/fileexchange/45134-violin-plot). Briefly, the violin plot function uses a Gaussian kernel to estimate the probability density of the data points. ‘Wider’ areas of the violin plot contain more individual observations (Fig. 4C, F).

### Single-unit, linear regression

**Figure 5:**
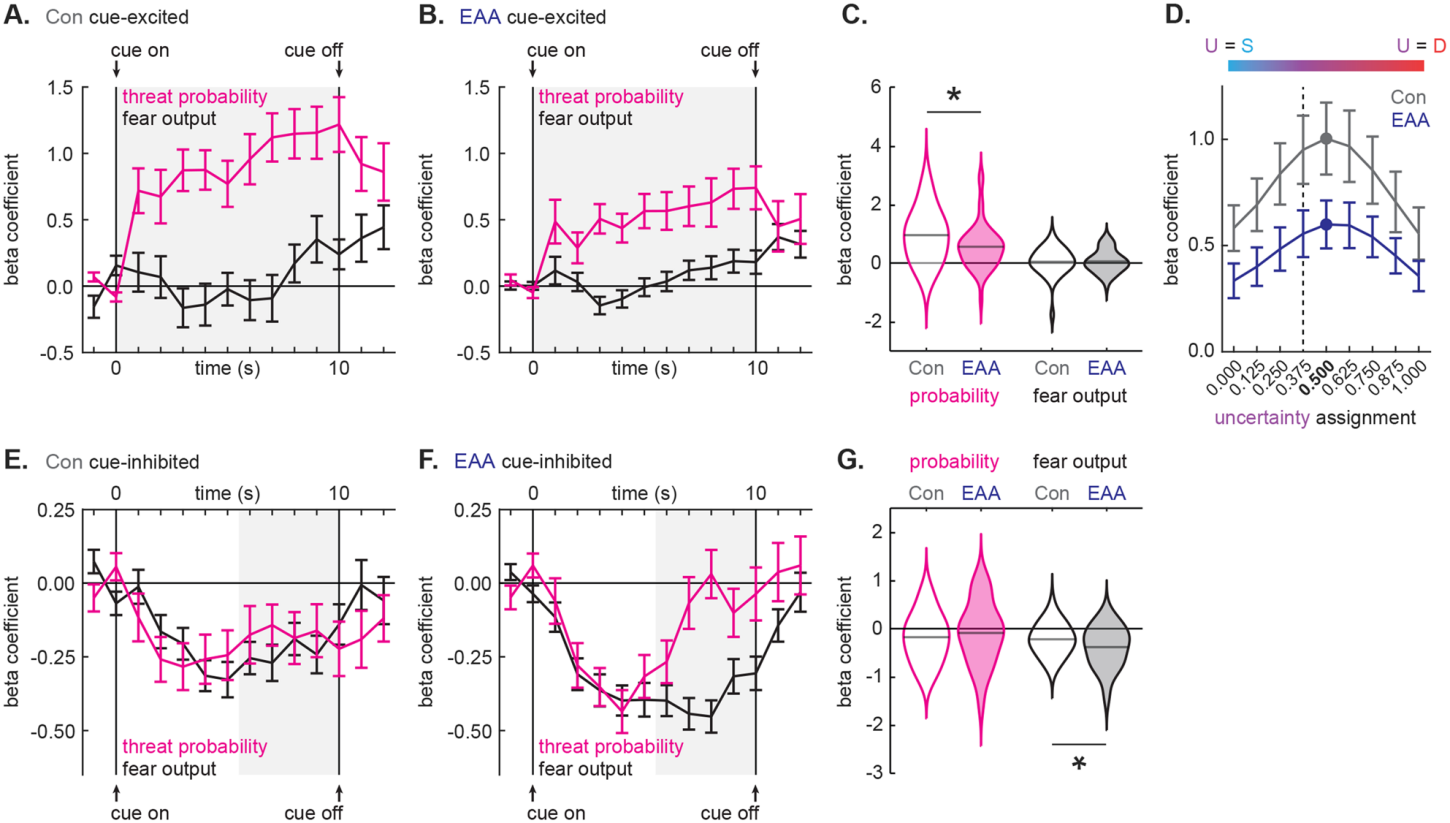
Altered threat probability and fear output signaling in EAA single-units. (**A**, **B**) Mean ± SEM beta coefficient is shown for threat probability (pink), and fear output (black) regressors during each 1 s cue interval for (**A**) Con cue-excited (n = 32) and (**B**) EAA cue-excited (n = 49) neurons. Cue onset (on) and offset (off) are indicated by vertical black lines. Light gray boxes indicate interval analyzed in (**C**). (**C**) Violin plot of beta coefficient is shown for each regressor, during the entire 10 s cue presentation, for the cue-excited population in Con (open) and EAA (closed). Mean beta coefficient is indicated by gray horizontal line. *Significant difference in beta coefficient for threat probability between Con and EAA (t_66_ = 2.71, p = 0.01; independent samples *t* test). (**D**) Mean ± SEM beta coefficient for threat probability is shown for each of the nine uncertainty assignments for Con cue-excited (gray) and EAA cue-excited (dark blue) neurons. The threat-tuning curve for Con and EAA cue-excited neurons peaked at an uncertainty assignment of 0.500, rather than the actual foot shock probability associated with uncertainty (0.375, dashed line). Color scale for uncertainty assignments is shown on top of the threat-tuning curve. (**E**, **F**) Mean ± SEM beta coefficient is shown for threat probability (pink), and fear output (black) during each 1 s cue interval for (**E**) Con cue-inhibited (n = 48) and (**F**) EAA cue-inhibited (n = 84) neurons. Light gray boxes indicate interval analyzed in (**G**). (**G**) Violin plot of beta coefficient is shown for each regressor, during the last, 5 s cue interval, for the cue-inhibited population in Con (open) and EAA (closed). Mean beta coefficient is indicated by gray horizontal line. *Significant difference in beta coefficient for fear output between Con and EAA (t_130_ = 2.31, p = 0.02; independent samples *t* test).

Single-unit, linear regression was used to determine the degree to which fear output and/or threat probability explained trial-by-trial variation in firing of single neurons in a specific time interval. For each regression, all 32 trials from a single session were ordered by type. Z score normalized firing rate was specified for the interval of interest. The fear output regressor was the suppression ratio for the entire cue, for that specific trial. The threat probability regressor was the foot shock probability associated with the specific cue. Regression (using the regress function in Matlab) required a separate, constant input. The regression output of greatest interest was the beta coefficient for each regressor (fear output and threat probability), quantifying the strength (greater distance from zero = stronger) and direction (> 0 = positive) of the predictive relationship between each regressor and single-unit firing. ANOVA, two-tailed dependent samples *t* test, and Pearson’s correlation coefficient were all used to analyze beta coefficients, exactly as described for normalized firing rate. Distribution of individual data points is visualized with violin plots (Fig. 5).

### Threat probability tuning curve

Nine separate regression analyses were performed as above. Only now, the value assigned to uncertainty component of the threat probability regressor was systematically increased from 0 to 1 in 0.125 steps (0.000, 0.125, 0.250, 0.375, 0.500, 0.625, 0.750, 0.875 and 1.000). The first regression used the value of 0.000, second regression 0.125 and so on. Regression was performed for each 1 s interval of the 10 s cue. Beta coefficients for all 10 intervals were averaged to produce a single threat tuning curve (Fig. 5D).

### Firing/signaling relationships

**Figure 6:**
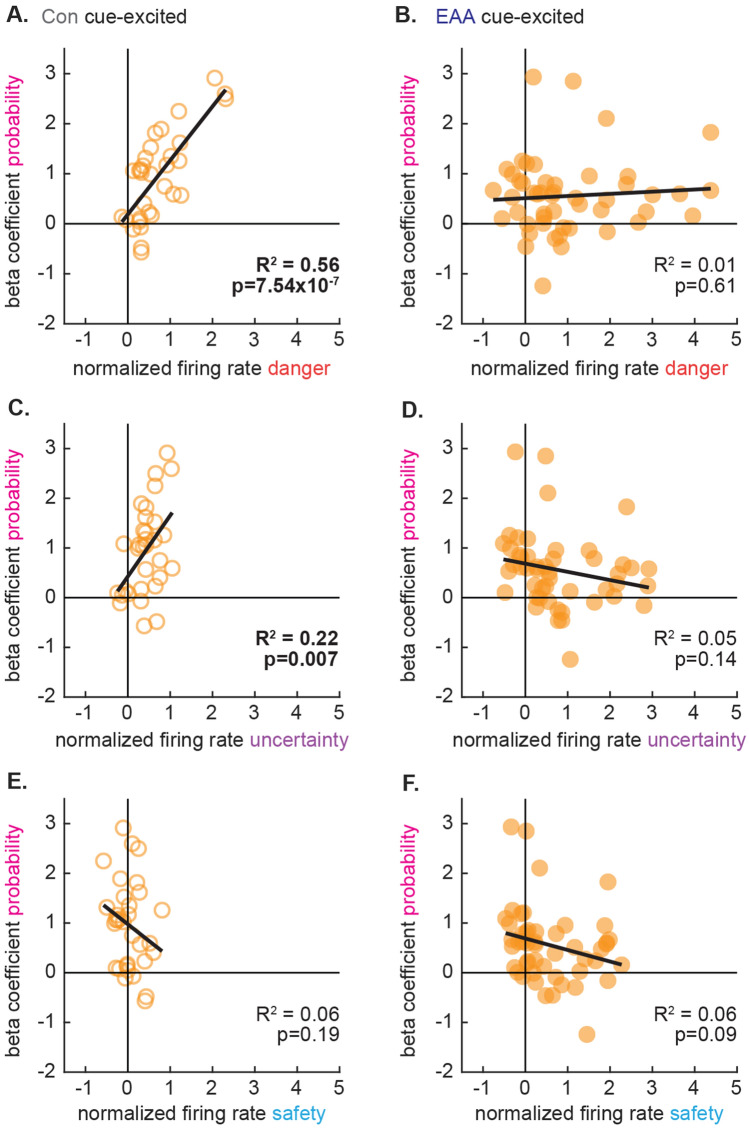
Cue responding and threat probability signaling decoupled in EAA cue-excited single-units. (**A**, **B**) Normalized firing rate (Z score) to danger during the first 1 s of cue presentation is plotted against mean beta coefficient for threat probability for (**A**) Con cue-excited (n = 32, open) and (**B**) EAA cue-excited (n = 49, closed) neurons. The trendline, square of the Pearson correlation coefficient (R^2^), and associated p value are shown for each plot. Identical plots made for uncertainty (**C**, **D**) and safety (**E**, **F**).

**Figure 7:**
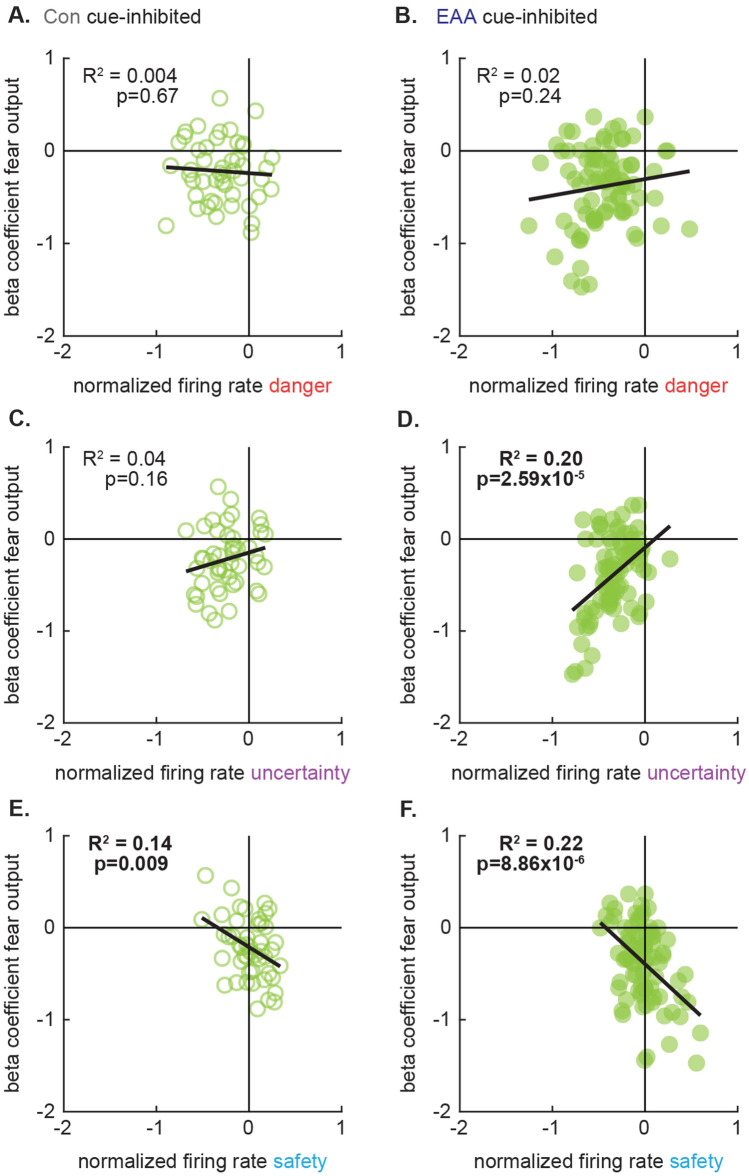
Cue responding and fear output signaling coupled in EAA cue-inhibited single-units. (**A**, **B**) Normalized firing rate (Z score) to danger during the last 5 s of cue presentation is plotted against beta coefficient for fear output for (**A**) Con cue-inhibited (n = 48, open) and (**B**) EAA cue-inhibited (n = 84, closed) neurons. The trendline, square of the Pearson correlation coefficient (R^2^), and associated p value are shown for each plot. Identical plots made for uncertainty (**C**, **D**) and safety (**E**, **F**).

The relationship between single-unit firing and information signaling were determined by plotting normalized firing rate (Z score) against the beta coefficient. This was done for each group (Con vs. EAA) and functional population [cue-excited (Fig. 6) vs. cue-inhibited (Fig. 7)] and was further done for each regressor (fear output and threat probability). Trendline, R^2^ and p value for the Pearson’s correlation coefficient are reported. Between group comparisons (Con vs. EAA) for predictive relationships were made using the Fisher R-to-z transformation https://vassarstats.net/rdiff.html.

### Data and software availability

Full electrophysiology data set will be uploaded to https://crcns.org/ upon acceptance for publication.

### Additional resources

Med Associates programs used for behavior and Matlab programs used for behavioral analyses are made freely available at our lab website: https://mcdannaldlab.org/resources.

## Results

Timed, pregnant Long Evans rats arrived on gestational day 14–16 and gave birth in the Boston College animal facility (Fig. 1A). Pups were weaned, sexed and single-housed on postnatal day (P) 21. Food and water were freely available at all times during adolescence. From P26 to P35, 12 female Long Evans rats underwent the adversity procedures^30,31^ in which four different stressors were given on five separate occasions over 10 consecutive days, AM and PM each day (Fig. 1A). Adverse experiences consisted of tail pinch, restraint, forced cold swim and cat hair exposure. Rats receiving early adolescent adversity (EAA) are referred to as the EAA group. Control rats (8 Long Evans females) were handled each day but did not receive adverse experiences. Consistent with prior reports^45,46^, our adversity procedures effectively slowed body weight gain (Fig. 1B). Body weights were equivalent prior to adversity, weight gain slowed in EAA rats, but body weights were equivalent between groups in adulthood. Analysis of variance (ANOVA) for body weights [repeated measures: day (14); factor: group (Con vs. EAA)], found a significant group × day interaction (F_13,234_ = 4.20, p = 3.00 × 10^–6^, partial eta squared (η_p_^2^) = 0.19, observed power (op) = 1.00). Supporting the interaction, control rats gained significantly more weight from P24 to P35 than did EAA rats. Independent samples *t* test for body weight change found a significant difference between groups (t_18_ = 6.11, p = 9.00 × 10^–6^; Fig. 1C). At the conclusion of the adversity procedure, rats remained single-housed and matured to adulthood with no further adverse experiences.

On P56, rats were food restricted and maintained at 85% of their free feeding body weight. Behavioral testing took place in experimental chambers consisting of a grid floor, central port and food cup. Rats were trained to nose poke in the central port in order to receive a food pellet from a cup below. Fear discrimination took place over a baseline of rewarded nose poking, but the schedules for nose poking and cue presentation were completely independent. During fear discrimination, three distinct auditory cues predicted a unique foot shock probability: danger (p = 1.00), uncertainty (p = 0.375), and safety (p = 0.00) (Fig. 1D). Trial order was randomized for each rat, each session. Fear was measured with suppression ratio and was calculated by comparing nose poke rates during baseline and cue periods (see “Methods”)^29–31,39,40,47,48^. Suppression ratios near one indicate high fear; near zero indicate low fear and intermediate levels indicate intermediate fear.

Control and EAA rats acquired discrimination over the initial 8 sessions, showing high fear to danger, lesser fear to uncertainty, and least fear to safety. EAA rats acquired discrimination faster than Controls, but discrimination levels were equivalent between groups by the final discrimination session (Fig. 1E, F). ANOVA for suppression ratios for all discrimination sessions (1–8) [repeated measures: session (8); factors: group (Con vs. EAA) and cue (danger vs. uncertainty vs. safety)], found a group × cue × session interaction (F_14,252_ = 1.99, p = 0.02, η_p_^2^ = 0.10, op = 0.95). ANOVA restricted to the final discrimination session found a main effect of cue (F_2,36_ = 151.45, p = 2.97 × 10^–18^, η_p_^2^ = 0.89, op = 1.00), but no group × cue interaction (F_2,36_ = 1.55, p = 0.23, η_p_^2^ = 0.08, op = 0.31).

Drivable microelectrode bundles were implanted just dorsal to the vlPAG (Fig. 2A). Following recovery from surgery, rats were returned to fear discrimination and single-unit activity was collected. Single-units were isolated at the start of each recording session and held for the session duration. The microelectrode bundle was advanced ~ 42–84 µm between sessions. Recording began on ~ P90 and went to P143 at the latest. Thus, any perturbations in vlPAG/DR function would have endured for 55–108 days following the conclusion of adversity.

### Higher proportion of EAA cue-responsive neurons

We recorded the activity of 151 neurons in 8 Control rats over 72 fear discrimination sessions, and 203 neurons in 12 EAA rats over 107 sessions (see Supplementary Fig. S1 online). Control and EAA rats showed equivalent baseline nose poke rates (t_18_ = 1.24, p = 0.23; Fig. 2B) and comparable fear discrimination during recording sessions: high fear to danger, lesser to uncertainty, and least to safety (Fig. 2C). ANOVA for mean suppression ratios [factors: group (Con vs. EAA) and cue (danger vs. uncertainty vs. safety)], found a main effect of cue (F_2,36_ = 199.47, p = 3.33 × 10^–20^, η_p_^2^ = 0.92, op = 1.00), but no group × cue interaction (F_2,36_ = 0.24, p = 0.79, η_p_^2^ = 0.01, op = 0.08). Paired samples *t* tests confirmed differing ratios for each group and cue (danger vs. uncertainty; uncertainty vs. safety; all t > 3.00, all p ≤ 0.01).

Previous work has identified cue-excited^20–22,24^ and cue-inhibited neurons^21,25^ as the two broad, functional vlPAG neuron types. We identified 32/151 Control neurons (21.2%) and 49/203 EAA neurons (24.1%) that were cue-excited, showing phasic increases in firing to danger, uncertainty, or safety (dependent samples *t* test for firing rate, baseline [10 s prior to cue onset] vs. first 1 s cue interval or baseline vs. last 5 s cue interval; p < 0.0083, corrected for six tests). We identified 48/151 Control neurons (31.8%) and 84/203 EAA neurons (41.4%) that were cue-inhibited, showing phasic decreases in firing to danger, uncertainty, or safety (dependent samples *t* test for firing rate, baseline [10 s prior to cue onset] vs. first 5 s cue interval or baseline vs. last 5 s cue interval; p < 0.0083, corrected for six tests). Combining the cue-excited and cue-inhibited populations in each group revealed a higher proportion of EAA cue-responsive neurons (133/203, 65.5%) compared to Controls (80/151, 53.0%); χ^2^ = 5.68, p = 0.02; Fig. 2D).

Baseline firing is used a proxy for vlPAG cell types, with GABA interneurons typically showing high baseline firing and glutamatergic output neurons showing low baseline firing^21^. The baseline firing rates of the single-units comprising the cue-excited and cue-inhibited populations, as well as single-units outside the selection criteria, were similar in Control and EAA rats (Fig. 2E, F). Independent samples *t* tests found no differences in baseline firing between Control and EAA cue-excited, cue-inhibited and neurons outside the selection criteria (all t < 1.10, all p > 0.25). By contrast, one-way ANOVA for the three functional neuron types, collapsing across Control and EAA groups found a main effect of functional type (F_2,346_ = 7.35, p = 0.001). Baseline firing rates were lowest for cue-excited neurons. Observing comparable baseline firing helps minimize the concern that differences in responding between Control and EAA neurons are due to sampling different cell types.

### Inflated cue responses in EAA neurons

While single-units were selected for general cue-responsiveness, differential firing was observed in all populations (Fig. 3). Control, cue-excited neurons scaled their firing on initial cue presentation: danger > uncertainty > safety. Danger firing was maintained, and cellular discrimination between danger, uncertainty and safety was observed for the entirety of cue presentation (Fig. 4A). EAA cue-excited neurons showed inflated firing on initial cue presentation (Fig. 4B) and selective firing for the remainder of cue presentation. Critically, the temporal pattern of selective firing differed for Control and EAA cue-excited neurons. ANOVA for normalized firing rate [data from Fig. 4A, B; factors: group (Con vs. EAA), cue (danger, uncertainty and safety) and bin (250 ms bins, 2 s prior to cue onset → 2 s following cue offset)] revealed a group × cue × bin interaction (F_110,8360_ = 3.32, p = 3.13 × 10^–28^, η_p_^2^ = 0.04, op = 1.00). The interaction held when bregma recording level was included as an ANOVA factor (F_110,8360_ = 1.49, p = 0.001, η_p_^2^ = 0.02, op = 1.00). The firing pattern for each population was observed on every trial (see Supplementary Fig. S2 online). Consistent with inflated responses to initial cue presentation, ANOVA for the first 1 s cue interval revealed a main effect of group (F_1,79_ = 4.96, p = 0.03, η_p_^2^ = 0.06, op = 0.60). Compared to Controls, EAA neurons showed excessive firing to safety (Bonferroni-corrected, independent samples *t* test, t_79_ = 3.61, p = 0.001; Fig. 4C). Yet in the first 1 s of cue presentation, Control and EAA cue-excited neurons each showed cellular discrimination between danger and uncertainty (Bonferroni-corrected, paired samples *t* test, all t > 2.50, all p < 0.025), as well as uncertainty and safety (all t > 3.00, all p < 0.025). So although cue responding was inflated, EAA did not disrupt the overall capacity of cue-excited neurons to discriminate danger, uncertainty and safety.

Control, cue-inhibited neurons showed equivalent decreases in firing to danger and uncertainty, but little change in firing to safety (Fig. 4D). This firing pattern was sustained for the entirety of cue presentation. Transient excitation at cue onset was driven by a handful of neurons showing rapid excitation to danger, followed by sustained inhibition (Fig. 3A). EAA cue-inhibited neurons showed enhanced decreases in firing to danger, lesser decreases in firing to uncertainty and little change to safety (Fig. 4E). This pattern was most apparent in the first half of cue presentation, with danger and uncertainty firing returning to baseline thereafter. Confirming different temporal firing patterns for Control and EAA neurons, ANOVA for normalized firing rate [data from Fig. 4D, E; factors: group (Con vs. EAA), cue (danger, uncertainty and safety) and bin (250 ms bins, 2 s prior to cue onset → 2 s following cue offset)] revealed a group × cue × bin interaction (F_110,14190_ = 1.69, p = 9.00 × 10^–6^, η_p_^2^ = 0.01, op = 1.00). The interaction was observed when bregma recording level was included as an ANOVA factor (F_110,13970_ = 1.34, p = 0.01, η_p_^2^ = 0.01, op = 1.00) and the firing pattern for each population was observed on every trial (see Supplementary Fig. S3 online). Supporting an interaction, EAA cue-inhibited neurons (Bonferroni-corrected, paired samples *t* test, t_83_ = 3.14, p = 0.002), but not Control cue-inhibited neurons (t_47_ = 1.40, p = 0.17), showed cellular discrimination between danger and uncertainty in the first half of cue presentation. In the same cue period, EAA cue-inhibited neurons also showed greater decreases in firing to danger compared to Control neurons (Bonferroni-corrected, independent samples *t* test, t_130_ = 2.70, p = 0.008; Fig. 4F). The group difference in firing to uncertainty fell short of the Bonferroni-corrected p value (t_130_ = 2.38, p = 0.019). Finally, ANOVA for mean firing over the first 5 s of cue presentation revealed a main effect of group (F_1,130_ = 10.82, p = 0.001, η_p_^2^ = 0.08, op = 0.90), indicating greater overall reductions in firing in EAA cue-inhibited neurons.

Analysis of firing reveals that adverse adolescent experience reshapes neural responses to threat-related cues in adult, vlPAG/DR neurons. EAA cue-excited neurons show inflated responses to cue presentation, with over-responding particularly demonstrated to safety. EAA cue-inhibited neurons show excessive decreases in activity to danger, and only these neurons fully discriminate uncertainty from danger. Of course, the observed differences in firing do not necessitate that EAA alters information processing by vlPAG/DR neurons.

### Diminished threat probability signaling in EAA cue-excited neurons

A previous study from our laboratory found that cue-excited neurons signal threat probability, rather than fear output, in male rats^24^. We first sought to determine whether threat probability signaling was observed in Control, cue-excited neurons from female rats. To do this, we used simultaneous linear regression for single-unit firing (Fig. 5). For each single-unit, we calculated the normalized firing rate for each trial (32 total trials: 6 danger, 6 uncertainty shock, 10 uncertainty omission, and 10 safety) in 1 s bins over the 10 s cue. Fear output (suppression ratio for 10 s cue presentation) was calculated for each trial and the corresponding threat probability was assigned: danger: 1.00, uncertainty: 0.375 and safety: 0.00. Fear output and threat probability were used as regressors to explain trial-by-trial variance in single-unit firing. The non-linear relationship between the probability of shock for uncertainty and the level of fear it elicited (Fig. 2C), permitted the two to be dissociated. Regression output for each single-unit was a beta coefficient for each regressor, quantifying the strength (|> 0| = stronger) and direction (> 0 = positive) of the predictive relationship. Beta coefficients were subjected to ANOVA with group (Control vs. EAA), regressor (fear output vs. threat probability) and interval (1 s intervals for 10 s cue) as factors.

Linear regression revealed that Control, cue-excited neurons signal threat probability (Fig. 5A). Beta coefficients were large and positive for threat probability at cue onset and increased over cue presentation. Beta coefficients for fear output were around zero for the majority of cue presentation, only modestly increasing toward the end of the cue presentation. EAA cue-excited neurons showed diminished threat probability signaling (Fig. 5B). Beta coefficients for threat probability were consistently lower for EAA cue-excited neurons than for Control cue-excited neurons, while beta coefficients for fear output were similar. Supporting this interpretation, ANOVA for beta coefficients [data from Fig. 5A, B repeated measures: 1 s cue interval (10); factors: group (Con vs. EAA) and regressor (fear output vs. threat probability)] revealed a group × regressor interaction (F_1,65_ = 6.80, p = 0.01, η_p_^2^ = 0.10, op = 0.73). The mean beta coefficient for the entirety of cue presentation differed for threat probability (independent samples *t* test, t_66_ = 2.71, p = 0.01), but not fear output (t_66_ = 0.17, p = 0.86), between Control and EAA cue-excited neurons (Fig. 5C).

Single-unit regression used the actual shock probability associated with uncertainty (0.375). However, subjects, and by extension vlPAG/DR neurons, did not have explicit knowledge of the actual shock probability. It is therefore possible that cue-excited neurons are ‘tuned’ to an alternative probability. It is further possible that early adversity alters the adult shape of this tuning curve, rather generally reducing threat probability signaling. To examine these possibilities, we performed single-unit linear regression maintaining the probabilities for danger (1.00) and safety (0.00), but increasing the assigned uncertainty probability from 0 to 1 in 0.125 increments for each analysis: 0.000, 0.125, 0.250, 0.375, 0.500, 0.625, 0.750, 0.875, to 1.000. The mean beta coefficient for each regression/increment is plotted as a threat-tuning curve for Control and EAA cue-excited neurons (Fig. 5D).

The threat-tuning curve for Control cue-excited neurons peaked at an uncertainty assignment of 0.500, rather than 0.375. Thus, threat-tuning in female vlPAG/DR neurons does not precisely match actual probability. As uncertainty assignment moved away from 0.500, beta coefficients dropped off. The tuning curve for EAA cue-excited neurons was identical in shape, but was diminished across all assignments. ANOVA for beta coefficients [data from Fig. 5D; repeated measures: assignment (9); factor: group (Con vs. EAA)] revealed a main effect of assignment (F_8,624_ = 18.88, p = 1.71 × 10^–25^, η_p_^2^ = 0.20, op = 1.00), but most critically a main effect of group (F_1,78_ = 4.01, p = 0.049, η_p_^2^ = 0.05, op = 0.51) and no group × assignment interaction (F_8,624_ = 1.25, p = 0.27, η_p_^2^ = 0.02, op = 0.58). Importantly, using the peak uncertainty assignment for linear regression returned an identical pattern of threat probability signaling as did the actual uncertainty assignment (see Supplementary Fig. S4 online).

### Enhanced fear output signaling in EAA cue-inhibited neurons

Linear regression revealed that Control, cue-inhibited neurons signaled a mix of threat probability and fear output (Fig. 5E). Beta coefficients were negative for each regressor for the majority of cue presentation. EAA cue-inhibited neurons also showed mixed signaling of threat probability and fear output in the first half of cue presentation. Signals diverged thereafter, with fear output persisting and threat probability diminishing (Fig. 5F). ANOVA for beta coefficients [data from Fig. 5E, F; repeated measures: 1 s cue interval (10); factors: group (Con vs. EAA) and regressor (fear output vs. threat probability)] revealed a group × regressor × interval interaction (F_9,1170_ = 2.14, p = 0.02, η_p_^2^ = 0.02, op = 0.89). Mean beta coefficient for fear output was more negative in EAA cue-inhibited neurons compared to Control neurons for the last half of cue presentation (independent samples *t* test, t_130_ = 2.31, p = 0.02; Fig. 5G). Beta coefficients for threat probability in the same period did not differ (t_130_ = 0.81, p = 0.42). An identical pattern of fear output signaling was observed for Control and EAA cue-inhibited neurons if the peak uncertainty assignment was used instead of the actual uncertainty assignment (see Supplementary Fig. S4 online).

Adverse adolescent experiences not only reshape neural responses to threat, but alter information processing in adult, vlPAG/DR neurons. EAA cue-excited neurons contained less information about threat probability. EAA cue-inhibited neurons show superior signaling of fear output, at the expense of signaling threat probability. Although cue firing and information signaling were analyzed separately, one would anticipate these are related in normal rats. Adverse adolescent experiences may not independently alter cue firing and information signaling, but alter the relationship between the two.

### Cue firing and threat probability signaling decoupled in EAA cue-excited neurons

We compared firing in the first 1 s of cue presentation (data from Fig. 4C) to threat probability signaling over the entirety of cue presentation (data from Fig. 5C) for control and EAA cue-excited neurons. Danger firing and threat probability signaling were coupled in Control cue-excited neurons such that greater danger firing at cue onset predicted greater threat probability beta coefficients for the cue duration (R^2^ = 0.56, p = 7.54 × 10^–7^; Fig. 6A). Danger firing and threat probability signaling were uncoupled in EAA cue-excited neurons, and there was zero predictive relationship between danger firing and threat probability signaling (R^2^ = 0.01, p = 0.61; Fig. 6B). Importantly, the relationship between danger firing and threat probability signaling significantly differed between Control and EAA cue-excited neurons (Fisher r-to-z transformation, Z = 3.80, p = 0.0001). An identical statistical pattern was observed for uncertainty firing and threat probability signaling. Control cue-excited neurons showed a positive relationship (R^2^ = 0.22, p = 0.007; Fig. 6C), EAA cue-excited neurons showed zero relationship (R^2^ = 0.05, p = 0.14; Fig. 6D) and these relationships significantly differed (Fisher r-to-z transformation, Z = 3.05, p = 0.0011). Safety firing and threat probability signaling were not related in either population, and this relationship did not differ between Control and EAA cue-excited neurons (Fig. 6E, F). Coupling in Control neurons and decoupling in EAA neurons was specific to threat probability signaling. An identical analysis comparing firing to fear output signaling found no predictive relationships for any cues, and no differences between these relationships for Control and EAA cue-excited neurons (see Supplementary Fig. S5 online).

### Cue firing and fear output signaling coupled in EAA cue-inhibited neurons

To determine whether similar decoupling was observed in cue-inhibited neurons, we compared firing and fear output signaling for the last half of cue presentation for control and EAA neurons. Degree of danger firing failed to predict fear output signaling in either population (Control: R^2^ = 0.004, p = 0.67; EAA: R^2^ = 0.02, p = 0.24; Fig. 7A, B) and these relationships did not differ (Fisher r-to-z transformation, Z = 1.04, p = 0.15). EAA cue-inhibited neurons, but not Control, showed a positive predictive relationship between uncertainty firing and fear output (Control: R^2^ = 0.04, p = 0.16; EAA: R^2^ = 0. 20, p = 2.59 × 10^–5^; Fig. 7C, D) but these relationships did not differ between groups (Fisher r-to-z transformation, Z = 1.43, p = 0.08). Equivalent negative predictive relationships between safety firing and fear output signaling were also found in both groups (Fisher r-to-z transformation, Z = 0.60, p = 0.54). Greater increases in firing to safety predicted larger, more negative beta coefficients for fear output (Control: R^2^ = 0.14, p = 0.009; EAA: R^2^ = 0. 22, p = 8.86 × 10^–6^; Fig. 7E, F). Cue firing was also predictive of threat probability signaling in both groups (see Supplementary Fig. S6 online), but there were no differences between Control and EAA cue-inhibited neurons for any predictive relationship. Early adversity sufficient to decouple firing and threat probability signaling in cue-excited neurons was insufficient to decouple firing and fear output signaling in cue-inhibited neurons.

## Discussion

We gave female, Long Evans rats early adolescent adversity (EAA) and recorded vlPAG/DR activity during multi-cue fear discrimination in adulthood, 50 + days following the final adverse experience. Despite achieving behavioral discrimination that was equivalent to Controls, threat-related responding was altered in EAA individuals. A greater proportion of EAA neurons were cue-responsive. Cue responding was inflated, with EAA cue-excited neurons showing greater firing increases and cue-inhibited neurons showing greater firing decreases compared to Controls. EAA cue-excited neurons showed reduced threat probability signaling, while cue-inhibited neurons showed enhanced fear output signaling. Coupling of cue responding and threat probability signaling was diminished in EAA cue-excited neurons. The results reveal long-lasting changes in vlPAG/DR threat responding following EAA.

Before considering broader implications, several factors should be considered. We deliberately chose female subjects. Childhood adversity increases adult risk for anxiety disorders in both sexes^1,2,49–51^, but this relationship may be more prevalent in females^52^. Examining the effects of EAA in female rats was the most logical starting point. Further, we have previous work showing greater effects of EAA on fear discrimination in females^31^. In that study, female EAA rats showed higher levels of conditioned suppression to uncertainty and safety. Using a procedure in which males show intermediate fear to uncertainty^24,25^, we observed much higher fear in females. This precluded the ability to detect adversity-induced increases. By contrast, Walker et al. 2018 used a foot shock probability of 0.25, and gave half as many trials per session. Using those experimental parameters, controls showed conditioned suppression to uncertainty that was intermediate to danger and safety. Making uncertainty (p = 0.375) more similar to danger, and providing a greater number of trials per session, may have lessened the difficulty of fear discrimination in the present study, mitigating the effect of early adversity. Walker et al. 2018 also sampled many more individuals per group (~ 14–16). While clear group differences were observed between Control and EAA subjects, there was overlap in behavioral performance among individuals that composed the two groups. Studies of early adversity and fear discrimination utilizing single-unit recordings with large sample sizes, though challenging, would permit greater understanding of individual differences in susceptibility and resilience to early adversity^53^.

Anatomically, our recordings spanned ~ 0.70 mm anterior–posterior and included the vlPAG and the DR. A previous study used an identical recording approach and more exclusively targeted the vlPAG^24^. Despite these differences, here we report highly similar patterns of single-unit firing to the previous study, as well as identical patterns of threat probability and fear output signaling. This may mean that single-units were primarily obtained from the vlPAG or that similar patterns of activity/signaling are observed in the DR^54^. The critical comparisons in this study are between Control and EAA subjects/neurons. Observed differences in firing/signaling could then be due to differences in the neuron-types sampled between groups. Control and EAA neurons showed mostly identical waveform and firing characteristics (Fig. 2E, F) and though altered, the gross firing patterns found in each group/neuron-type were similar (Fig. 4). Differences in overall fear discrimination could also have potentially accounted for differences in neural activity. However, Control and EAA subjects showed identical patterns of fear discrimination. While the necessity of a between-subjects design will present issues for any early adversity study, we feel those issues have been minimized here.

If early adversity can alter periaqueductal threat responding without disrupting adult fear discrimination, what it is the greater relationship between early experience, adult behavior and periaqueductal function? First, the relationship between early adversity and adult behavior is not absolute. Experiencing adversity does not guarantee impaired fear discrimination in adulthood. Even when we observe effects of adversity at the group level (n = 12–16), there is overlap in the fear discrimination abilities of the individuals^30,31^. Concerning the relationship between vlPAG responding and behavior, we find that vlPAG neurons normally show greater signaling of threat probability. The vlPAG activity reflecting fear output is normally only observed in cue-inhibited neurons^25^, and these neurons also signal threat probability. We interpret this to indicate that the vlPAG activity normally provides an estimate or prediction of impending threats, like that which is typically ascribed to the amygdala. A vlPAG threat estimate may be trained by the amygdala, but become more amygdala-independent following training. For example, inhibiting central amygdala inputs to the vlPAG has no effect on vlPAG onset responding to a fear conditioned cue, and only partially reduces late cue responding^20^.

At the same time, our results reveal the challenges ahead in devising therapies to restore adversity-induced ventral periaqueductal function. Strategies to broadly inhibit activity to threatening cues would ameliorate inflated responding by cue-excited neurons, but exacerbate responding by cue-inhibited neurons. Such therapies may have no effect on fear-related symptoms or even worsen them. Strategies to broadly excite activity would fail for similar reasons. Developing therapies to selectively target cue-inhibited and cue-excited populations will be challenging, given the apparent, heterogeneous cell-type composition of these functional populations. A more complete understanding of the connectivity and transcriptome of vlPAG/DR cell-types, as is underway for the DR^55,56^, will hopefully permit progress in this area. Revealing the normal developmental trajectory of vlPAG/DR neurons^57^—plasticity, synaptic transmission and connectivity—and its disruption by early adversity, will further specify targets for effective therapies.

Our results do not mean the vlPAG/DR should supplant the amygdala as the source of early adversity-induced threat dysfunction. Instead, we believe our results reveal that EAA must alter adult function in a multitude of regions across the larger fear circuit. Whether early adverse experience manifests in maladaptive fear behavior is likely to depend on a host of factors: biological sex, type/number of experiences, and adult behavior assessed. Additionally, the extent and the degree to which the larger neural circuit is compromised likely dictates the absence or presence of behavioral impairment. Studies simultaneously measuring threat-related activity across many brain regions, including periaqueductal gray and DR, will more rapidly detail the extent and degree to which fear circuits are compromised by EAA^58,59^.

## Supplementary information

Supplementary Figure S1.

Supplementary Figure S2.

Supplementary Figure S3.

Supplementary Figure S4.

Supplementary Figure S5.

Supplementary Figure S6.

Supplementary Information.
