# Supplementary figures and images for: Early adolescent adversity alters periaqueductal gray/dorsal raphe threat responding in adult female rats
Source: Sci Rep. 2020 Oct 22;10:18035. doi: 10.1038/s41598-020-74457-3 (PMC7582948; doi:10.1038/s41598-020-74457-3)

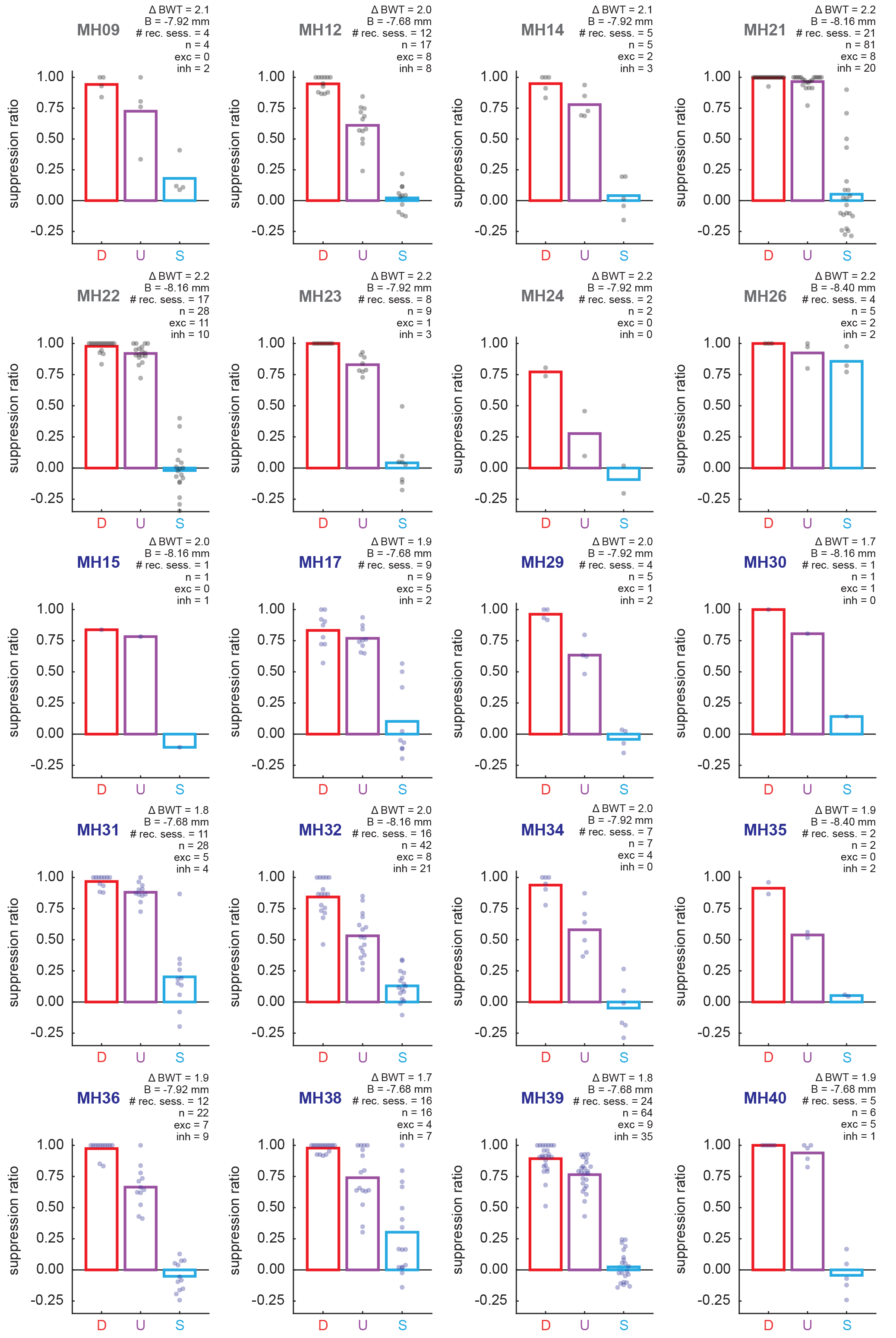

Supplement: Supplementary file 1 — Supplementary Figure S1. [file 41598_2020_74457_MOESM1_ESM.tif]

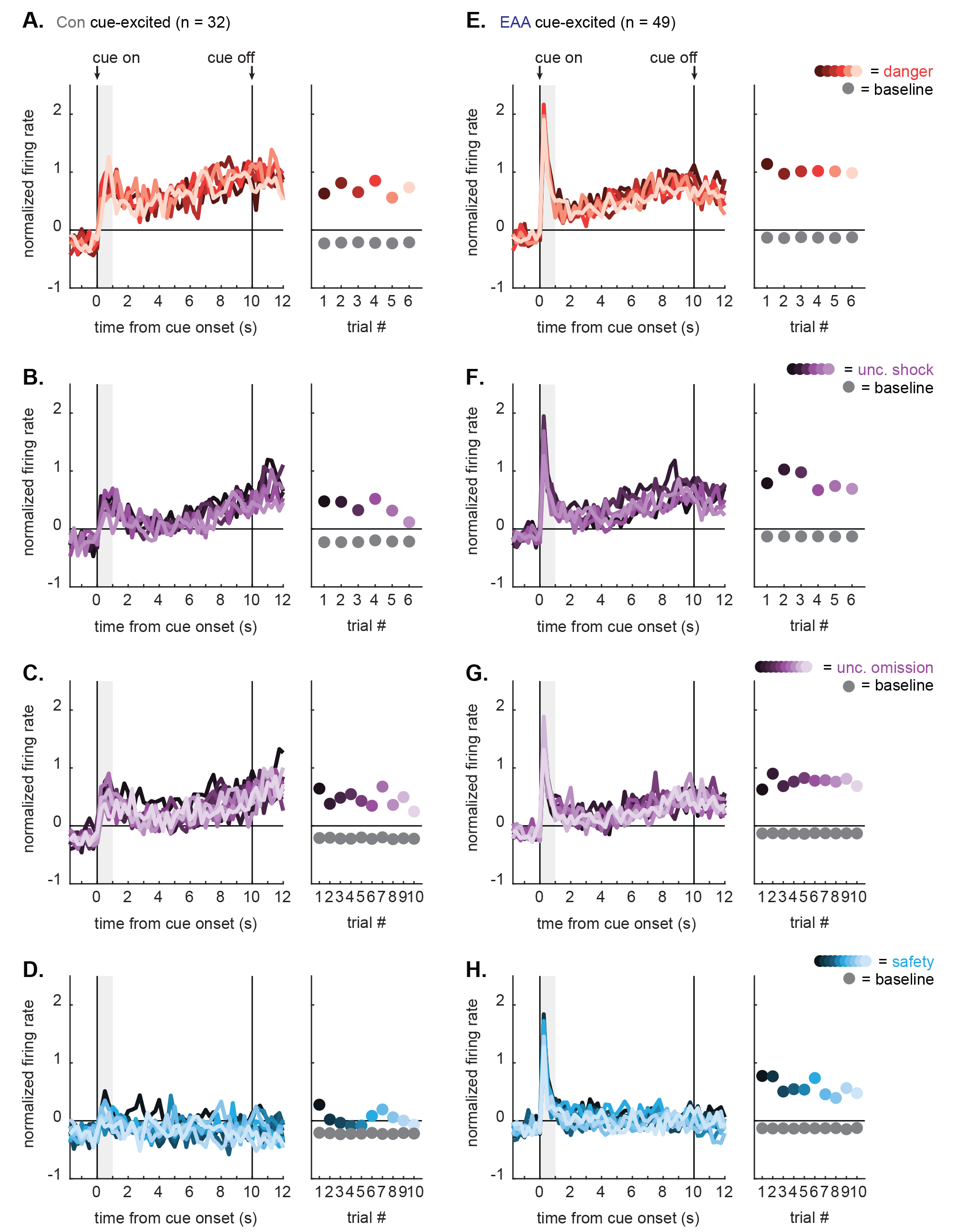

Supplement: Supplementary file 2 — Supplementary Figure S2. [file 41598_2020_74457_MOESM2_ESM.tif]

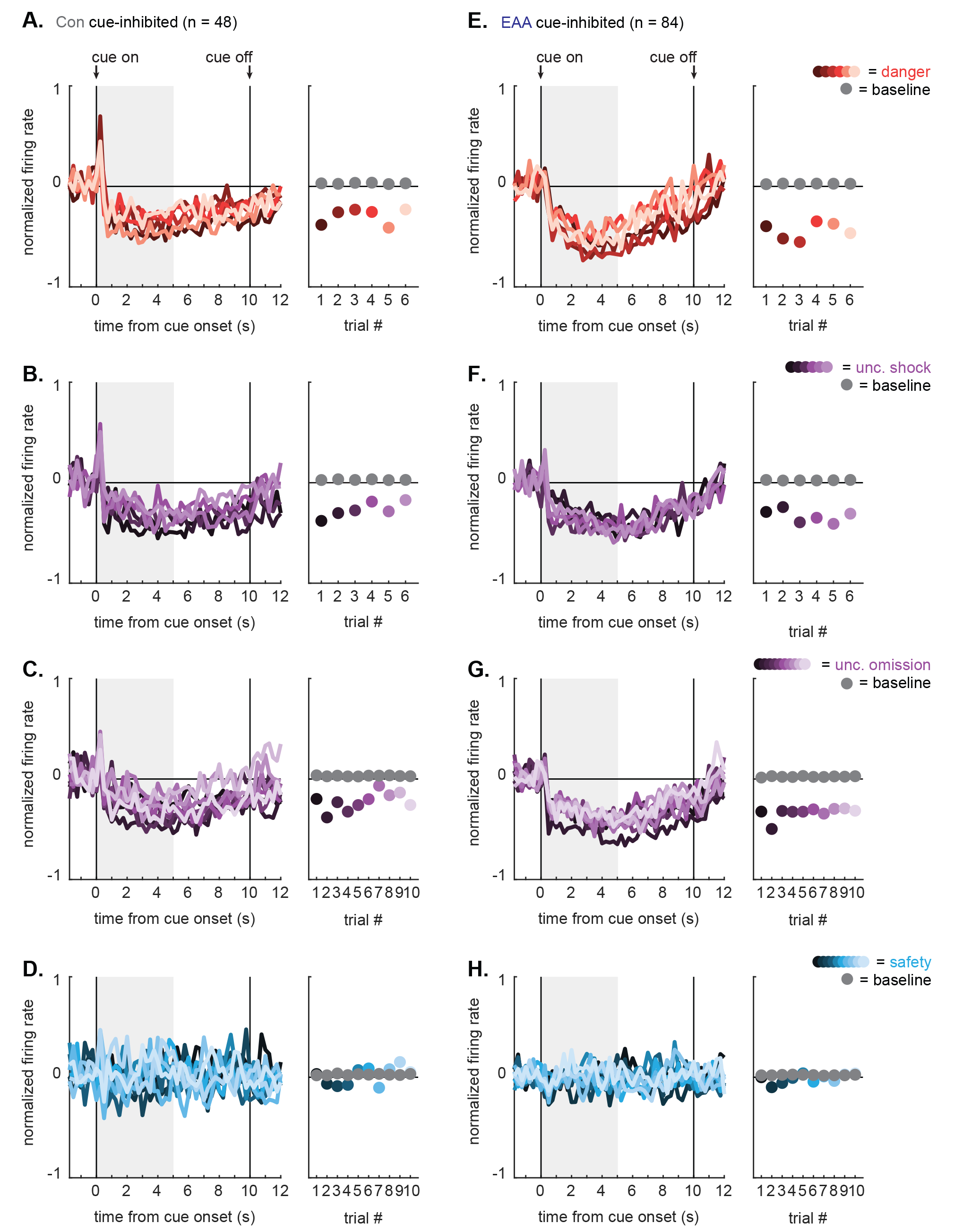

Supplement: Supplementary file 3 — Supplementary Figure S3. [file 41598_2020_74457_MOESM3_ESM.tif]

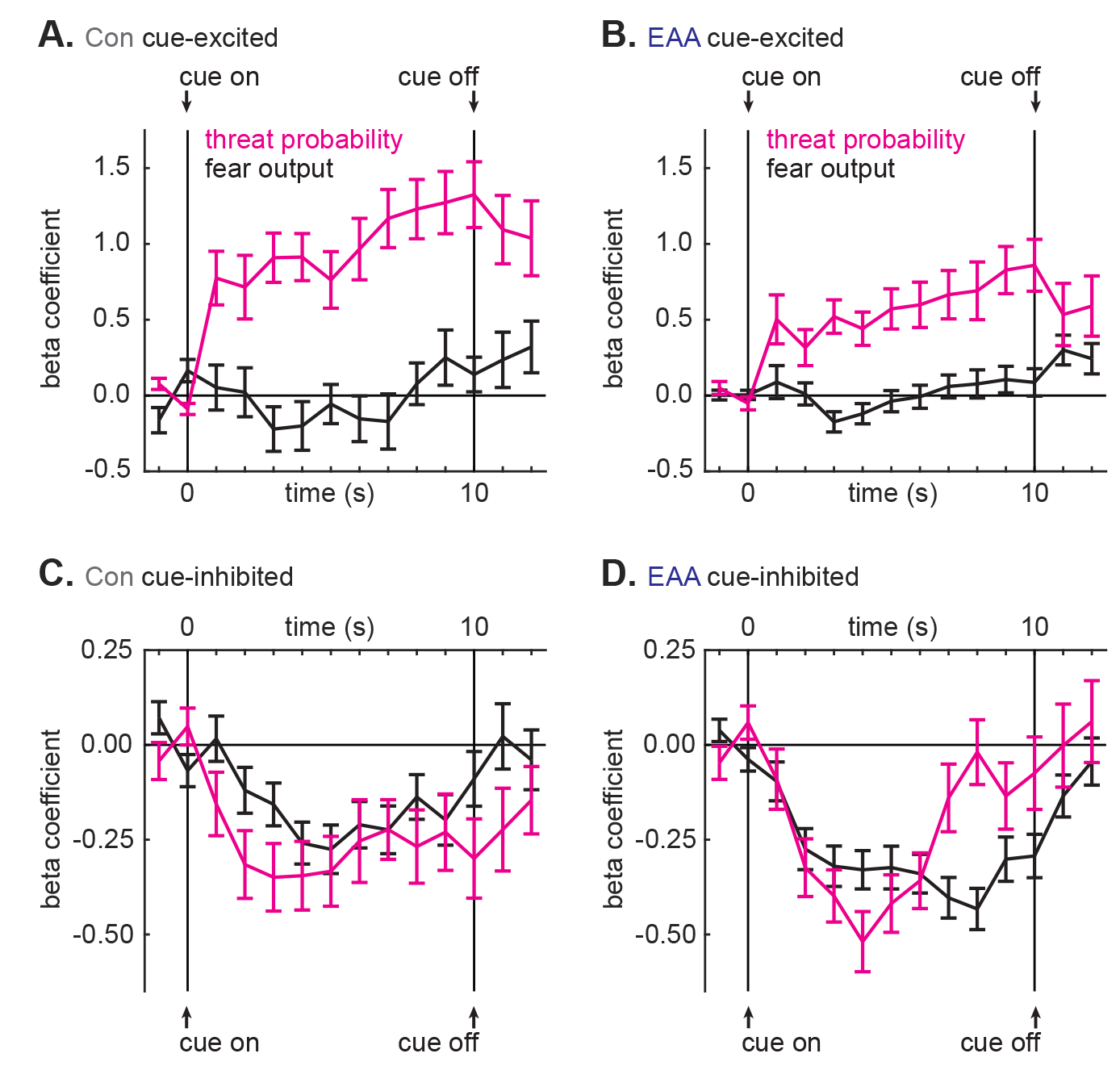

Supplement: Supplementary file 4 — Supplementary Figure S4. [file 41598_2020_74457_MOESM4_ESM.tif]

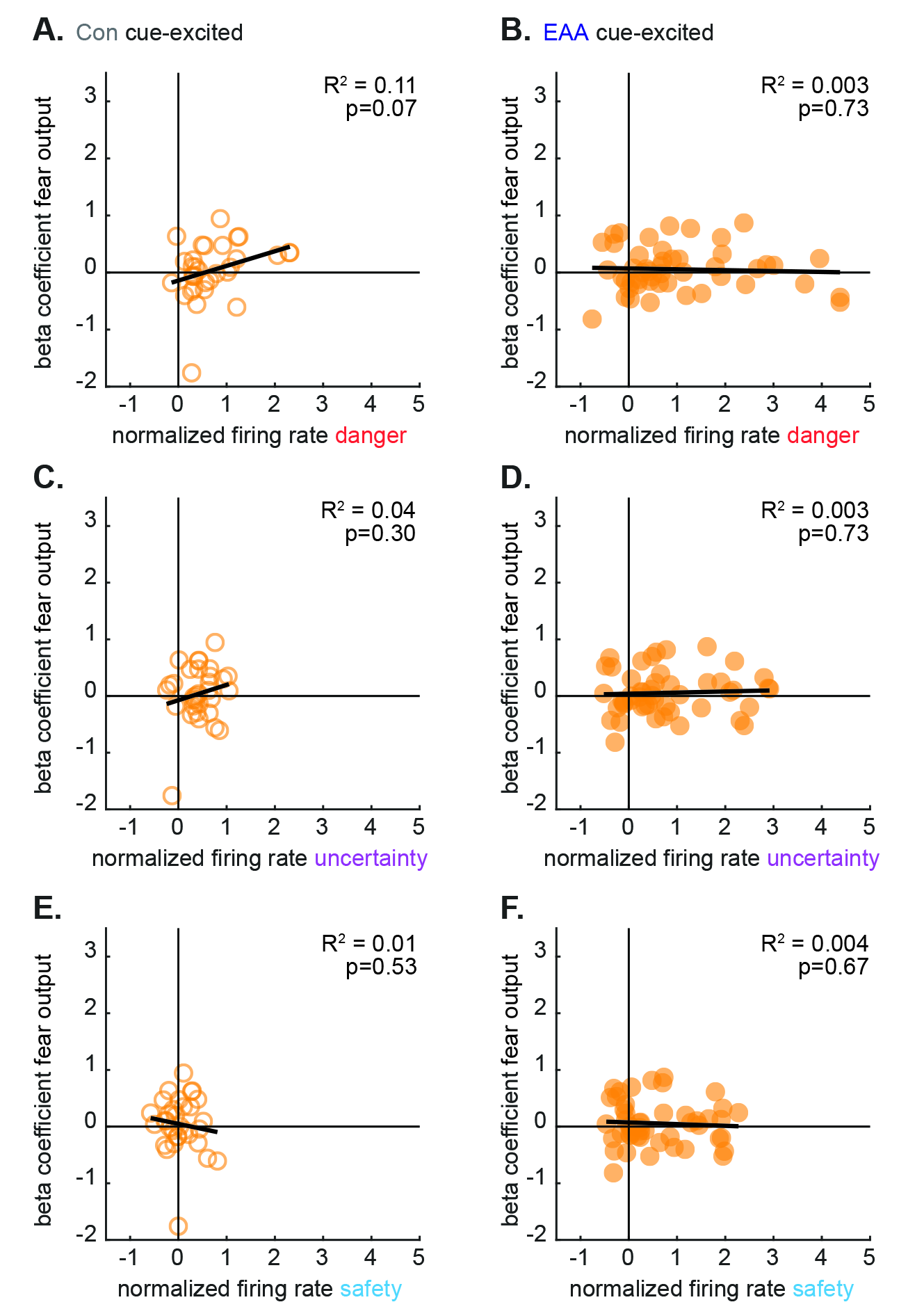

Supplement: Supplementary file 5 — Supplementary Figure S5. [file 41598_2020_74457_MOESM5_ESM.tif]

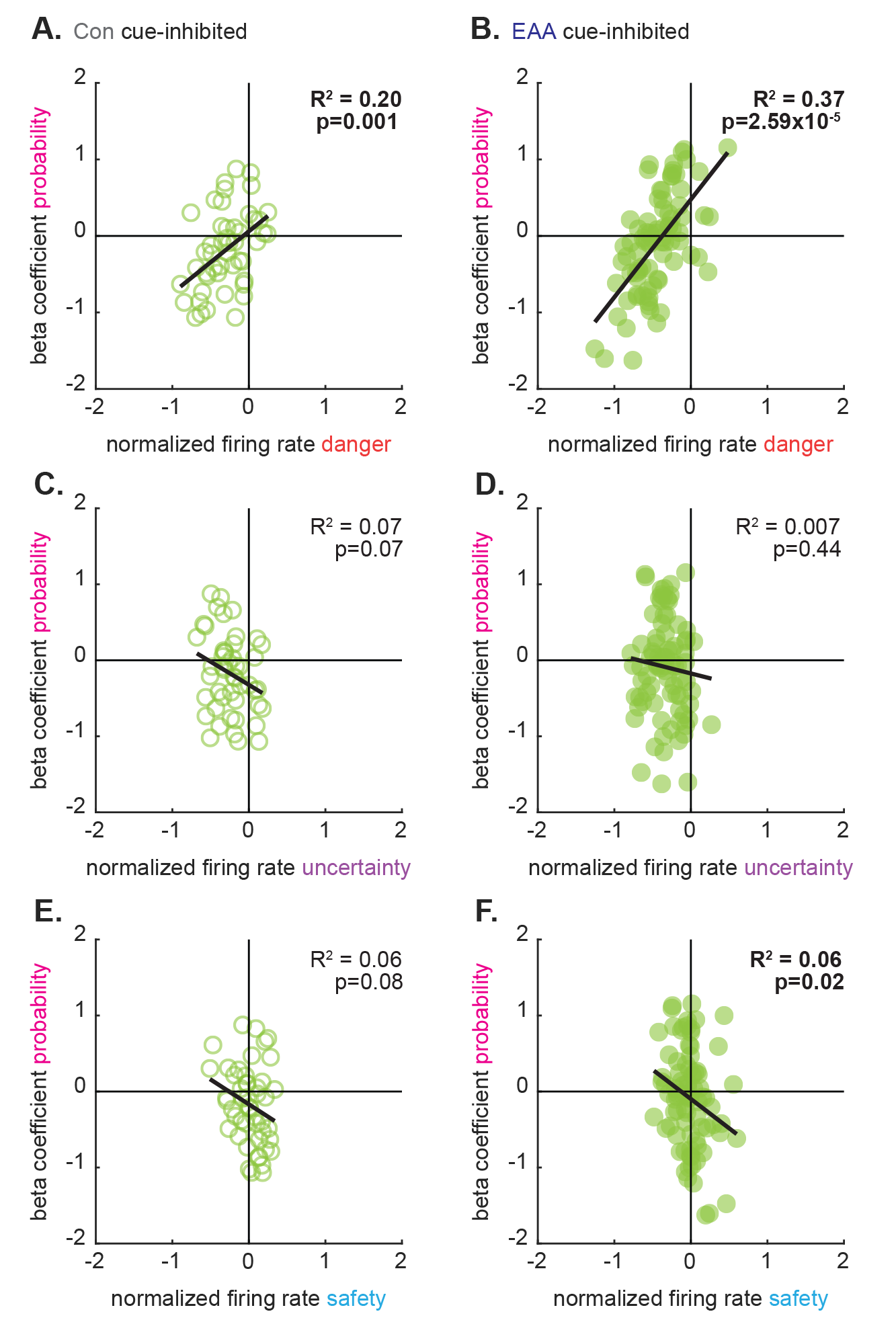

Supplement: Supplementary file 6 — Supplementary Figure S6. [file 41598_2020_74457_MOESM6_ESM.tif]
