# Supplementary material for: Early adolescent adversity alters periaqueductal gray/dorsal raphe threat responding in adult female rats
Source: Sci Rep. 2020 Oct 22;10:18035. doi: 10.1038/s41598-020-74457-3 (PMC7582948; doi:10.1038/s41598-020-74457-3)
Supplement: Supplementary file 7 — Supplementary Information. [file 41598_2020_74457_MOESM7_ESM.docx]

**Title**

**Early adolescent adversity alters periaqueductal gray/dorsal raphe threat responding in adult female rats**

**Running title**

Adversity and periaqueductal threat responding

**Author names and affiliations**

Mahsa Moaddab Ph.D., Kristina M. Wright M.A. and Michael A. McDannald Ph.D.*

*Lead contact

Boston College

Department of Psychology and Neuroscience

140 Commonwealth Ave.

514 McGuinn Hall

Chestnut Hill, MA 02467

Please address correspondence to M.M. ([moaddab@bc.edu](mailto:moaddab@bc.edu)) or M.A.M. ([michael.mcdannald@bc.edu](mailto:michael.mcdannald@bc.edu)).

**Supplemental information titles and legends**

**Figure S1.** Fear discrimination levels of all Control and EAA individuals.

Mean suppression ratio during the entire 10 s cue for danger (D, red), uncertainty (U, purple), and safety (S, blue) trials is shown for each individual for all sessions in which single-units were recorded. Session data points are superimposed on individual means for Con (n = 8, gray data points) and EAA (n = 12, dark blue data points). For each individual, the change in body weight (Δ BWT, p35/p24), bregma recording level (B), number of recording sessions, number of recorded neurons, and number of neurons in each category (cue-excited, exc; cue-inhibited, inh) are provided.

**Figure S2.** Trial-by-trial firing for cue-excited population.

(**A**, left) Normalized firing rate (Z score) for Con cue-excited neurons (n = 32) is shown across cue presentation for each of the six danger trials. Trials are color-coded from dark (first trial) to light (last trial). Cue onset (on) and offset (off) are indicated by vertical black lines. (**A**, right) Population mean firing rate is shown for the first 1 s cue interval (light gray box in A), for each trial. Color scheme maintained from (A, left). Population mean baseline firing rate (2 s prior to cue presentation) is shown for each individual trial (gray circles). Identical plots using unique color schemes were made for each trial type: (**B**) uncertainty shock (six trials, purple), (**C**) uncertainty omission (ten trials, purple), and (**D**) safety (ten trials, blue). (**E**-**H**) Identical set of plots were made for EAA cue-excited neurons (n = 49). Firing patterns observed for the mean of all trials (Fig. 4A, B) were observed at the single trial level for Con cue-excited and EAA cue-excited neurons.

**Figure S3.** Trial-by-trial firing for cue-inhibited population.

(**A**, left) Normalized firing rate (Z score) for Con cue-inhibited neurons (n = 48) is shown across cue presentation for each of the six danger trials. Trials are color-coded from dark (first trial) to light (last trial). Cue onset (on) and offset (off) are indicated by vertical black lines. (**A**, right) Population mean firing rate is shown for the first 5 s cue interval (light gray box in A), for each trial. Color scheme maintained from (A, left). Population mean baseline firing rate (2 s prior to cue presentation) is shown for each individual trial (gray circles). Identical plots using unique color schemes were made for each trial type: (**B**) uncertainty shock (six trials, purple), (**C**) uncertainty omission (ten trials, purple), and (**D**) safety (ten trials, blue). (**E**-**H**) Identical set of plots were made for EAA cue-inhibited neurons (n = 84). Firing patterns observed for the mean of all trials (Fig. 4D, E) were observed at the single trial level for Con cue-inhibited and EAA cue-inhibited neurons.

**Figure S4.** Alternative threat probability signaling in vlPAG/DR single-units.

Linear regression was performed for cue-excited and cue-inhibited neurons exactly as described in the manuscript, but using an uncertainty assignment of 0.500 (peak of the tuning curve), rather than 0.375 (actual foot shock probability). (**A** and **B**) Mean ± SEM beta coefficient is shown for regressors; threat probability (pink), and fear output (black) during each 1 s cue interval for (A) Con cue-excited (n = 32) and (B) EAA cue-excited (n = 49) neurons. (**C** and **D**) Identical plots were made for (C) Con cue-inhibited (n = 48) and (D) EAA cue-inhibited (n = 84) neurons.

**Figure S5.** Cue responding and fear output signaling were not related in either Control cue-excited or EAA cue-excited neurons.

(**A** and **B**) Normalized firing rate to danger during the first 1 s of cue presentation is plotted against beta coefficient for fear output over the entire 10 s cue presentation for (A) Con cue-excited (n = 32, open) and (B) EAA cue-excited (n = 49, closed) neurons. Trendline, the square of the Pearson correlation coefficient (R^2^), and p value are shown for each plot. Identical plots made for uncertainty (**C** and **D**) and safety (**E** and **F**).

**Figure S6.** Cue responding and threat probability signaling were coupled in both Control cue-inhibited and EAA cue-inhibited neurons.

(**A** and **B**) Normalized firing rate to danger is plotted against beta coefficient for threat probability during the last 5 s of cue presentation for (**A**) Con cue-inhibited (n = 48, open) and (**B**) EAA cue-inhibited (n = 84, closed) neurons. Trendline, the square of the Pearson correlation coefficient (R^2^), and p value are shown for each plot. Identical plots made for uncertainty (**C** and **D**) and safety (**E** and **F**).
